# Verification of Selected Failure Criteria for Adhesive Bonded Elements with Different Stiffness through the Use of Methacrylic Adhesive

**DOI:** 10.3390/ma13184011

**Published:** 2020-09-10

**Authors:** Paweł Maćkowiak, Bogdan Ligaj, Dominika Płaczek, Maciej Kotyk

**Affiliations:** Faculty of Mechanical Engineering, University of Science and Technology, 85-796 Bydgoszcz, Poland; bogdan.ligaj@utp.edu.pl (B.L.); dominika.placzek@utp.edu.pl (D.P.); maciej.kotyk@utp.edu.pl (M.K.)

**Keywords:** methacrylic adhesive, adhesive joints, single-lap joint, strength criterion, stress distributions

## Abstract

This study presents the testing results of methacrylic adhesive single-lap joints made from elements with different stiffness and of the adhesive itself, using cast specimens. Methods for the preparation and testing of material specimens of the adhesive joints have been presented. Moreover, an attempt was undertaken to determine the strength criterion and find out which of the presented calculation methods enables the most precise assessment of strength in the tested group of single-lap joints, that differ in terms of the adhered stiffness and thickness. For this purpose, C45 steel and 5754 aluminium flat bars were bonded. Stress distributions were determined for failure forces obtained in the experiment by means of three basic analytic and numerical methods. Stress and strain states were compared, indicating the highest consistency for the value of normal peel stresses acting in the direction perpendicular to the direction of the joint tension. Reduced stresses provided by the analyses reached values higher than those which were achieved during the specimen tension testing.

## 1. Introduction

Adhesive joints are getting more and more popular. Their primary advantage is that different types of materials can be bonded. Moreover, these joints are lightweight and less time is needed to prepare them. Due to these advantages, designers can enjoy more freedom in the selection of materials which, in turn, makes it possible to provide more lightweight materials in a shorter time [1,2]. This is confirmed by the wide application of adhesive joints, particularly in the automotive, aviation and biomedical industries.

The strength parameters of adhesive joints are determined on the basis of tests performed according to specific norms. Examples of experimental test results are included in the work carried out by [3,4]. Other non-standard research methods for adhesive joints are presented in the work carried out by [5,6]. They involve the application of a “sandwich”-type specimen which is subjected to four-point bending. The tests provide a stress distribution for specified areas of a specimen, as a function of its bending. This method is significantly different from a normative test of adhesive joints which uses lap joints. However, calculation of the joint strength is still a challenge. Analytic methods are complicated and limited to specific types of joints. Numerical methods provide more freedom in creating a joint geometry [7,8,9], however, it is necessary to use appropriate models and material data for the adhesive and the elements to be bonded. The mechanical properties of an adhesive can be obtained for cast specimens [10] or for specimens which are tested for the joint adhesive layer [11]. It is also necessary to accept a criterion in the form of a boundary value, the attainment of which means that the maximal, permitted loading of the joint has been achieved [12,13]. Failure criteria can be grouped into the following categories: maximum stress or strain criteria, critical stress or strain at a distance or over a zone, limit state criteria, fracture mechanics criteria and damage mechanics criteria [14]. Only the first three criteria find applications for static loads.

Maximal shear stress is a natural failure criterion used in lap joints [15,16,17]. In further studies, plastic strains and high strains occurring in joints have been considered [18]. Use of the level of normal peel stress as a failure criterion is another approach [19]. The maximum principal tensile stress and maximum principal tensile strain criteria were used by Harris and Adams to predict the failure of single-lap joints [18]. The application of maximum von Mises stress as a failure criterion for scarf joints of metal and composite adherends was not successful [20]. This is the maximal value of the considered failure parameter that poses a problem for all the described maximal stress and strain criteria, particularly when a numerical linear analysis is used. The mechanical properties of the adhesive and adherends differ significantly. A rapid change of joint stiffness occurs at the lap ends which causes the occurrence of a peculiarity [21]. Maximal stress significantly differs depending on the applied grid. Use of local approximations, to remove the peculiarity, shifts the problem into the category of choosing which approximation should be used so as not to affect the joint strength. The criteria which are supposed to solve the problem are based on the values of maximal stresses and strains that occur in the zone or at a certain distance from the peculiarity. The criterion of mean weighted stress at a distance from the lap end and equal to the joint thickness was presented by Zhao [22]. The value of stresses from the determined zone was averaged and compared with the value of the adhesive yield point. Clarke and McGregor proposed a criterion in which the maximum principal stress must exceed the ultimate tensile stress of the adhesive material over a finite zone, normal to the direction of maximum principal stress. The size of this zone is a property of the adhesive that can be determined from a combination of analysis and testing. The analysis of the joints revealed that the method was prone to a 15% error. Another approach is based on a comparison of strains that occur at a certain distance from the peculiarity, to the maximal strains of the adhesive specimen [23].

The above methods have a significant disadvantage which involves the necessity to carry out assessment testing of the considered zone size or the distance from the peculiarities and this depends on many factors.

Limit state criteria assume that a joint is able to carry increasing loads until the whole cross-section undergoes plasticisation [22,24,25].

A different approach is a failure model for single-lap joints based on finite fracture mechanics. Only two basic fracture parameters are required: the tensile strength and the fracture toughness of the adhesive. The failure loads predicted are always conservative and the largest error made is about 22% [26,27]. Coupled stress and energy are used to model crack initiation in the adhesive layer [28].

The goal of this study is to identify a criterion for the calculation of the failure force that destroys a single-lap joint in order to provide the most consistent results when elements of different stiffness are being connected. An additional aim of the work is to present the method and results of testing the material properties of the Plexus MA300 methacrylic adhesive and single-lap joints made with its use.

The scope of this study covers the determination of the mechanical properties of the adhesive and experimental determination of the forces that destroy single-lap joints of steel and aluminium flat bars of different thickness. The material tested was a two-component structural adhesive for joining metals, plastics and ceramics: Plexus MA300 methacrylic adhesive. Moreover, analytic calculations and FEM simulations were performed. An attempt was made to choose a method and a criterion to be used for assessment of the joint strength on the basis of the stress and strain values calculated for the experiment maximal forces without the necessity to perform experimental tests.

## 2. Materials and Methods

### 2.1. Materials

Methacrylic adhesive MA300 was used in single-lap joint specimens. Manufacutrer of this adhesive was ITW Performance Polymers located in Denvers, MA, USA. The adhesive under consideration was applied to structural joints in metals, plastics and composites. Table 1 shows the property specifications of the adhesive provided by the manufacturer. The bonded materials were 1.5 mm thick C45 flat bars made of steel and flat bars made of 5754 aluminium.

### 2.2. Methods

#### 2.2.1. Tests of Mechanical Properties of the Adhesive

**Figure 1:**
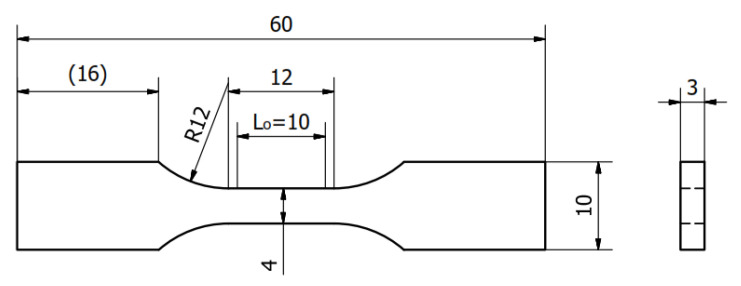
Dimensions in mm of the test specimens for determining mechanical properties under static tension in accordance with EN ISO 527-1.

The specimens used for the determination of static mechanical properties in tensile testing were designed in accordance with the EN ISO 527-1 standard [30], using dimensioning for the small 1BB shape. The dimensions of the specimens are shown in Figure 1.

**Figure 2:**
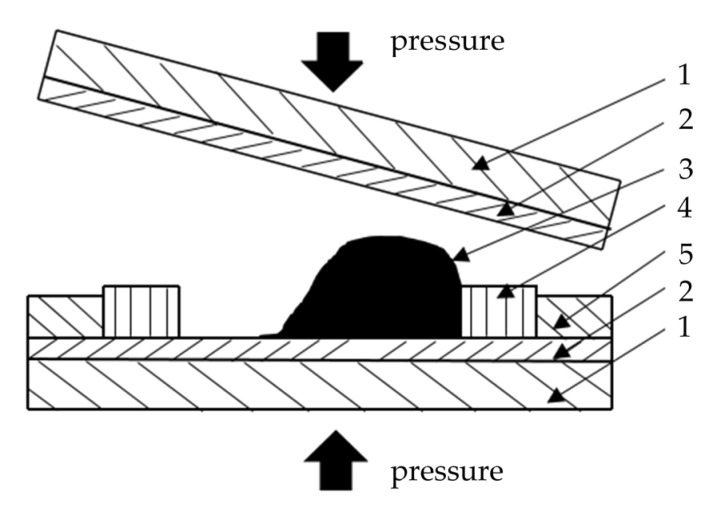
Structure of the mould and a method for casting methacrylic adhesive plates: 1—stiff plate, 2— polytetrafluoroethylene (PTFE) plate, 3—cast material, 4—silicone mould, 5—PTFE limiter.

Specimens of a methacrylic adhesive were made of cast plates with the dimensions 75 mm × 45 mm × 5 mm. A mixer, recommended by ITW Performance Polymers, was used for mixing two components of the adhesive. The paste adhesive was applied closer to one edge of the mould, and then the mould was closed at a slight angle to distribute the adhesive inside, as shown in Figure 2. This method of application reduces the risk of air bubbles in the casting. To avoid the moulded material sticking to the mould, its interior consisted of a polytetrafluoroethylene (PTFE) plate and a silicone frame. The clamp provided the cast plate with smooth lower and upper surfaces which made it possible to avoid its mechanical treatment. A silicone frame-shaped mould prevented excessive outflow of the adhesive from the mould. Additionally, it ensured sealing and the occurrence of overpressure. After curing, the cast plates of the methacrylic adhesive were removed from the moulds. Then, the target shape of the specimen was milled from the plate.

**Figure 3:**
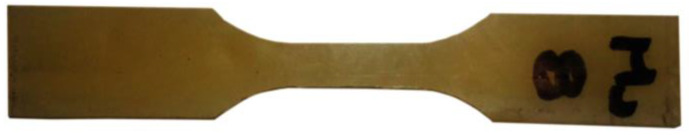
Physical form of the test specimen under static loading conditions.

The device presented in Figure 2 was used to prepare specimens from the Plexus MA300 adhesive with the dimensions presented in Figure 1. An example of specimen is presented in Figure 3. Specimens were seasoned for one month in room conditions (temperature 20 ± 2 °C, humidity 45 ± 5%).

**Figure 4:**
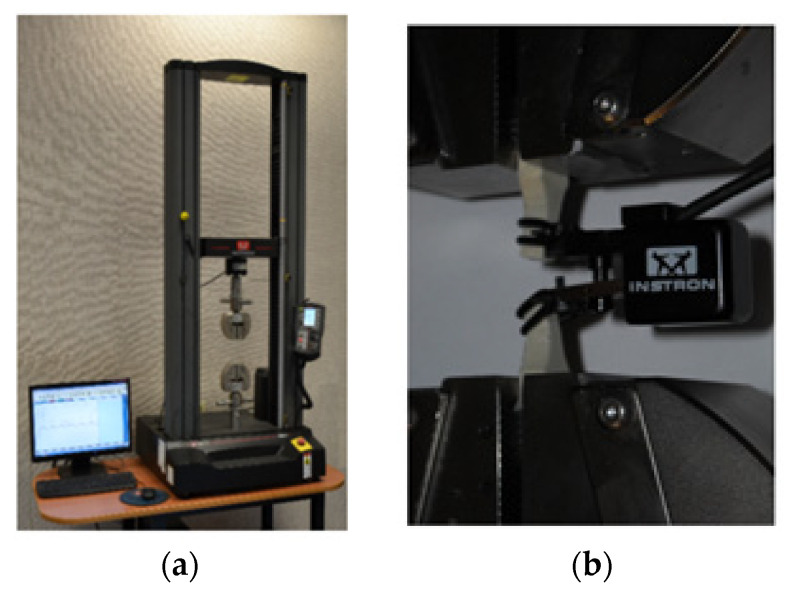
Test stand: (**a**) Instron 5966 testing machine, (**b**) tensile test and specimens in holders.

In the tensile testing, the specimens were fixed in a strength testing machine, with a device for force measurements up to 10 kN (Figure 4a). During the tests, the displacement parameter for a constant value was accepted to be equal to 0.005 mm·s^−1^ for both directions. Two extensometers were used during the tests. The first extensometer was used for measuring longitudinal strain, with a 10 mm measurement base and a measuring range of ±1 mm, as shown in Figure 4b. The second extensometer was used for measuring transverse deformations with an adjustable measuring base and measuring range of ± 0.5 mm.

#### 2.2.2. Tests of Mechanical Properties of Adherend Materials

**Figure 5:**
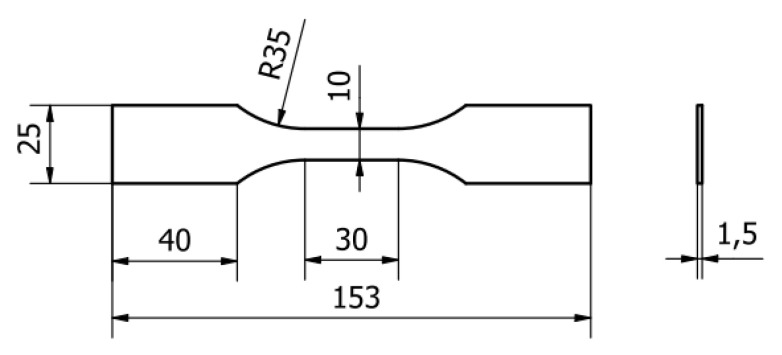
Dimensions in mm of the test specimens for testing the mechanical properties of adherend materials.

Tensile testing of the adherend material properties was carried out according to the PN-EN ISO 6892-1 norm [31]. The tests were controlled by traverse displacement, which was 0.05 mm/s., and were carried out using an INSTRON 8502 strength testing hydraulic machine (Instron, Norwood, MA, USA) with a dynamometer and measurement scope up to 200 kN. Strain was measured by means of a longitudinal extensometer fixed on the specimen, with a measurement base of 25 mm, and a transverse extensometer. Five specimens of each material were tested. The dimensions of the specimens are presented in the scheme in Figure 5.

#### 2.2.3. Tests of Single-Lap Joints

**Figure 6:**
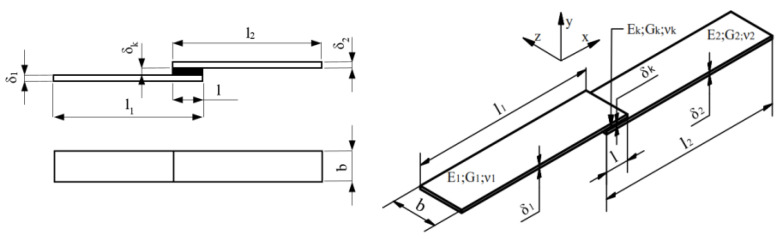
Dimensions of the single-lap specimens in accordance with PN-EN 1465-2009.

Specimens of single-lap adhesive joints were designed according to the PN-EN 1465-2009 norm [32]. The dimensions are presented in Figure 6. The adherends were flat bars made from 1.5, 3.0 and 5.0 mm thick C45 steel and aluminium. Five configurations of joints were tested, as presented in Table 2. A one-millimetre-thick layer of the Plexus MA300 adhesive was used to connect each element.

**Figure 7:**
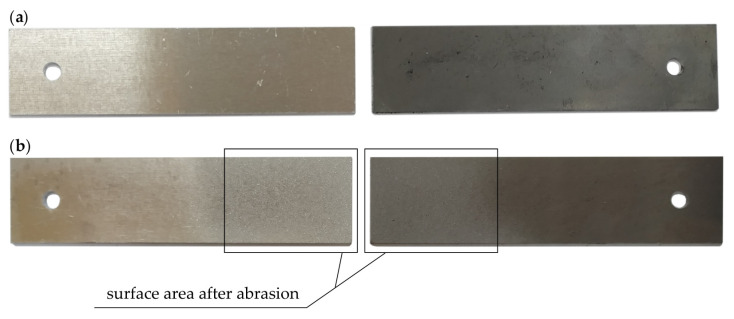
Flat bars to be used in tests: (**a**) accepted for tests, (**b**) after abrasion with electrocorundum.

Flat bars, with dimensions 100 mm × 25 mm × 1.5 mm, were cut out from steel sheets by a laser with target thicknesses (Figure 7). The works by [1,27,28] indicate that there is a significant impact of the surface preparation method on the strength and durability of the adhesive joints. The EN 13887:2003 [33] norm provides the methods for preparation of the surfaces of different materials. Prior to curing, the surface was prepared by dry abrasive blasting with electrocorundum, with an 80 µm grain size. Next, the surface was de-greased three times with acetone by the immersion method. It was wiped twice by a piece of cloth, until the dissolvent had evaporated. After the treatment, the mean value of the surface roughness was Ra = 2.55 μm, for steel, and Ra = 4.08 μm, for aluminium.

**Figure 8:**
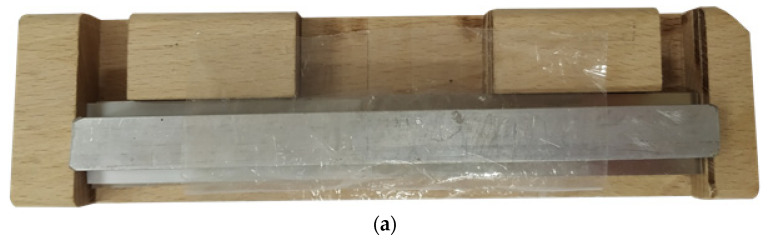

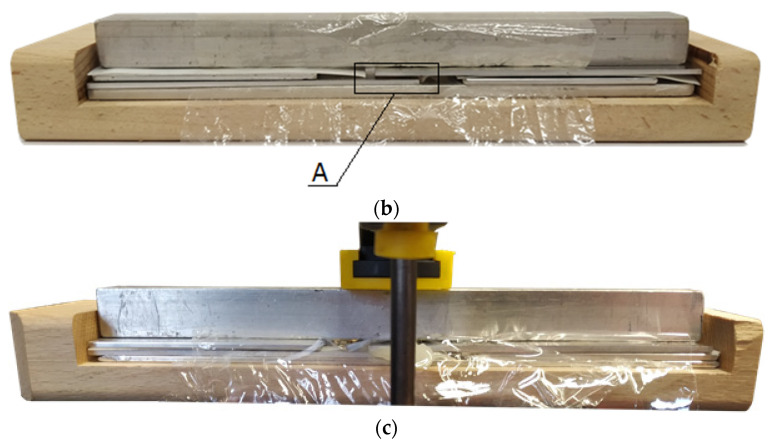
A test stand for adhesion of a single-lap specimen: (**a**) top view, (**b**) front view, (**c**) view of device during the process of the specimen adhesion; A—place for a joint.

Figure 8 shows a device for adhesion. The bonded flat bars were moved to the resistance surfaces of the device, adjusting the thickness of the joint by the application of PTFE distancers. Next, they were pressed from the top by a stiff beam and a carpenter clamp. In this way, specimens with repeatable dimensions and small parallelism deviation of the flat bars (in relation to each other) were obtained. Specimens were selected for testing on the basis of geometrical measurement results. Specimens with significant dimensional and geometric deviations were rejected.

**Figure 9:**
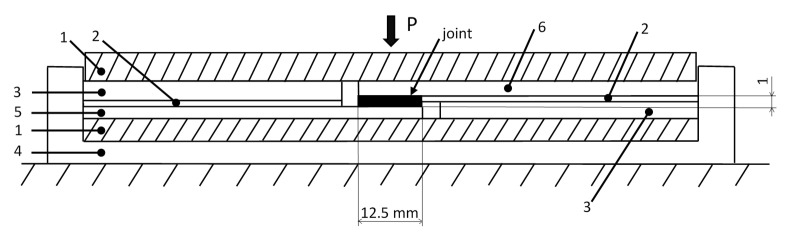
Scheme of a stand for adhesion of a single-lap specimen: 1, 3—steel divider, 2—PTFE divider, 4—base of the stand, 5,6—elements of the specimen, P—clamp force.

The stand consists of a wooden base (4) with dividers (1–3) and single parts of the specimen located between them (5,6) (Figure 9). The wooden base is used for the appropriate placement of the specimen. Moving single parts of the specimens toward particular surfaces on the base prevents their displacement in relation to each other and provides the adhesive single-lap joint with a repeatable dimension. The application of two dividers made of poly(tetrafluoroethylene) prevents adhesion of the specimen to the elements of the stand. Foil covers dividers 1 and 3 to provide an additional protective element. The thickness of dividers 2 and 3 determines the thickness of the adhesive joint. After the adhesion process, a clamp was added to the central point of the joint.

The dividers (1,2,3), parts of the specimen (5) and the protective foil were placed on the wooden base (4) prior to the adhesion process. Application of an adhesive on the joint was followed by: application of the second part of the specimen (6), spreading the foil, placing the divider (1) and joint pressing using a carpenter’s press. Figure 8c shows a single-lap specimen during bonding.

**Figure 10:**
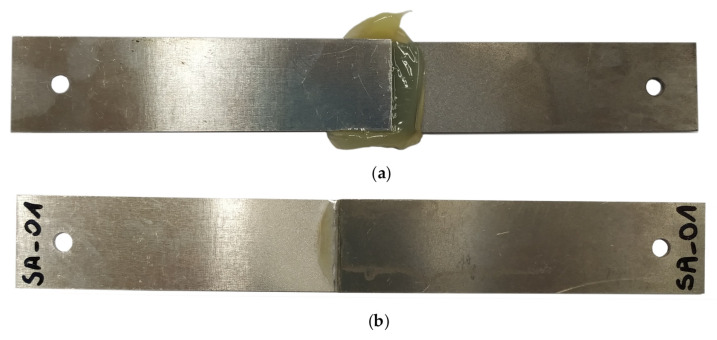
A single-lap specimen: (**a**) after being removed from the stand, (**b**) after removal of the adhesive scraps (test specimen).

Figure 10a shows the physical form of the specimen after its removal from the test stand. The specimen was subjected to treatment that involved removing scraps of the adhesive until the adhesive was present only on the surface of the lap, with dimensions 25 mm × 12.5 mm × 1 mm (Figure 10b).

**Figure 11:**
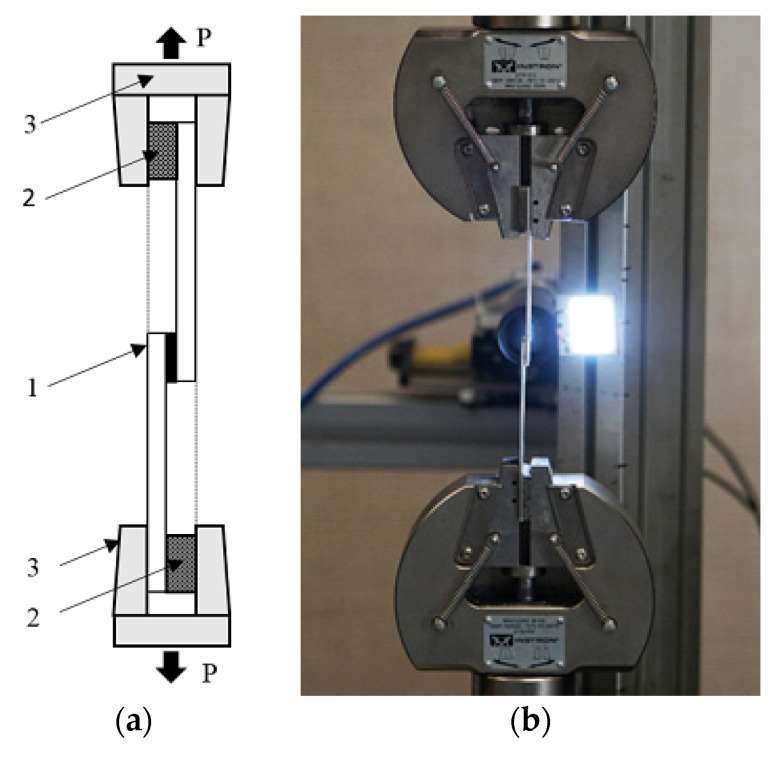
The way of the specimen montage in a holder of the strength testing machine: (**a**) scheme of montage: 1—specimen, 2—pad, 3—holder of the strength testing machine, (**b**) image of the test.

Single-lap specimens of adhesive joints were fixed in the holders of an INSTRON 5966 strength testing machine (Figure 11b, Instron, Norwood, MA, USA). Tests were conducted in room conditions: temperature 20 °C, moisture 55%. The specimen loading in the adhesive layer axis was obtained through the application of jaw pads (Figure 11a). The pad thickness was equal to the total thickness of the adhesive and the second adherend. The specimens were tested under the conditions of the machine piston displacement control with a speed equal to 0.05 mm/s. During the test, the values of force and the machine holder displacement were recorded.

Failure shear stresses are determined by their tension effect on single-lap adhesive joints. This calculation is performed by a simple engineering analytical method with an assumption of total stiffness, no strain of the adherend materials and zero thickness of the adhesive layer. Loading of the joint only causes strain in the adhesive layer. The value of shear stress τ in this method is described by dependence (Equation (1)):

(1) $$\tau =\frac{P}{b\times l}$$

#### 2.2.4. Analytic Methods for Determination of Stress Distributions in Single-Lap Joints

Volkersen [15] was the first to analytically determine the non-uniform distribution of shear stresses in an adhesive joint, with the following assumptions:

- Linear–plastic properties of the adherends and the adhesive joint;
- Uniform tension of adherends in each cross-section;
- Lack of the impact of the loading eccentricity causing bending of the adherends on the distribution of shear stresses in the adhesive joint.

The formula for adhesive joint shear stress distribution is described by a dependence given by Volkersen (Equation (2)):

(2) $$\tau \left(x\right)=\frac{\sigma_{01}\delta_{1}m}{\left(1+S\right)\sinh\left(ml\right)}\left[S\times \cosh\left(mx\right)+\cosh\left(m\left(l-x\right)\right)\right]$$

where

(3) $$m=\sqrt{\frac{G_{k}}{\delta_{k}}\times \frac{\delta_{1}E_{1}+\delta_{2}E_{2}}{\delta_{1}E_{1}\delta_{2}E_{}}}$$

(4) $$S=\frac{\delta_{1}E_{1}}{\delta_{2}E_{2}}$$

Goland and Reissner [16] took into account the impact of the misalignment of forces that load the lap joint through application of moment to the ends of the lap. The value of the moment is defined by using the bending moment coefficient *k*, given by (Equation (5))

(5) $$k=\frac{1}{1+2\sqrt{2}\tanh\frac{\lambda c}{2\sqrt{2}}}$$

where

(6) $$\lambda =\sqrt{12\left(1-\nu^{2}\right)}\times \frac{\sqrt{P^{\prime }tE^{\prime }}}{t}=\sqrt{\frac{P^{\prime }}{E^{\prime }{I^{\prime }}_{z}}}$$

c—the distance of the lap end from the joint centre.

The above solution requires acceptance of the following simplifications:

- Adherends are identical, in terms of material and shape;
- Width of the joint is significantly larger than its thickness;
- Thickness of the adhesive is negligibly small;
- Angle between the impact line of forces and the surface of adherends is small;
- Use of the theory of strain flat state is acceptable.

Taking into consideration the normal stresses σ_x_ acting in the direction of the applied force, shear stresses τ_x_ and omitting the remaining ones makes the applicability of the Goland–Reissner model limited by the following conditions (Equations (7) and (8)):

(7) $$\frac{\delta_{1}G_{k}}{\delta_{k}G_{1}}<0.1$$

(8) $$\frac{\delta_{1}E_{k}}{\delta_{k}E_{1}}<0.1$$

According to Goland–Reissner analysis, shear stress distribution is expressed by the dependence (Equation (9)):

(9) $$\tau \left(x\right)=-\frac{P}{4lb}\left\{\frac{8G_{k}}{E\delta_{1}\delta_{k}}\frac{1}{2}\left(1+3k\right)\frac{\cosh\sqrt{\frac{8G_{k}}{E\delta_{1}\delta_{k}}}x}{\sinh\sqrt{\frac{8G_{k}}{E\delta_{1}\delta_{k}}}\frac{1}{2}}+3\left(1-k\right)\right\}$$

Adams and Peppiatt proposed a method which, like Volkersen’s analysis, does not take into account the specimen bending, although it does consider the transverse area reduction of both adherends and the adhesive itself. The equations allow the determination, not only of normal and shear stresses along the length of a specimen, but also across its width [34,35]. Shear stress along the tension direction takes the form (Equation (10)):

(10) $$\tau \left(x\right)=-\frac{P\alpha }{b}\left\{\frac{\left[1-\psi \left(1-\cosh\left(\alpha l\right)\right)\cosh\left(\alpha x\right)\right]}{\sinh\left(\alpha l\right)}-\psi \sinh\left(\alpha x\right)\right\}$$

where

(11) $$\alpha =\sqrt{\frac{2G_{1}G_{2}G_{k}\left(E_{1}\delta_{1}+E_{2}\delta_{2}\right)}{E_{1}\delta_{1}E_{2}\delta_{2}\left(\delta_{1}G_{2}G_{k}+\delta_{2}G_{1}G_{k}+2\delta_{k}G_{1}G_{2}\right)}}$$

(12) $$\psi =\frac{E_{2}\delta_{2}}{E_{1}\delta_{1}+E_{2}\delta_{2}}$$

All of the analyses presented above assume that there is only elastic strain in the adhesive. As regards analytic methods, those that employ implicit equations can also be applied and the results are obtained by means of computer software. One such method is the Hart-Smith method. It assumes that the behaviour of the adhesive is of an elastic–plastic character, i.e., the value of stress on the ends of the laps stops rising after exceeding its maximum. The higher the loading, the longer the distance of stable, maximal loads.

Analytic methods have a complicated form and, additionally, they are based on a series of simplifications which increases the likelihood of a divergence between the calculation results and reality. Use of numerical methods is an alternative. An attempt to apply these methods is presented in this article.

#### 2.2.5. Numerical Method for the Determination of Stress Distribution in Lap Joints

**Figure 12:**
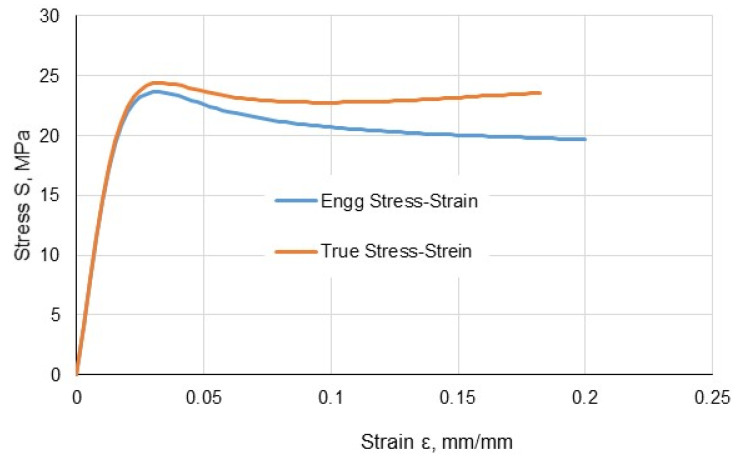
True stress as a function of true strain for methacrylic adhesive.

The calculations were performed using a two-dimensional analysis in the ABAQUS 6.6–4 software (Dassault Systèmes, Vélizy-Villacoublay, France). Adherends and the layer of an adhesive were modelled according to the dimensions of the lap specimens used for experimental tests. Material data obtained from the specimens’ tensile tests were assigned to the joint elements. Young’s modulus, the Poison coefficient and a true stress–true strain diagram were introduced into the program. Figure 12 shows an exemplary diagram for a methacrylic adhesive, showing the difference between the engineering diagram and the true stress–true strain diagram. The above data allowed us to carry out a non-linear analysis, including the plastic strain of materials and high strains that occur in elements.

**Figure 13:**
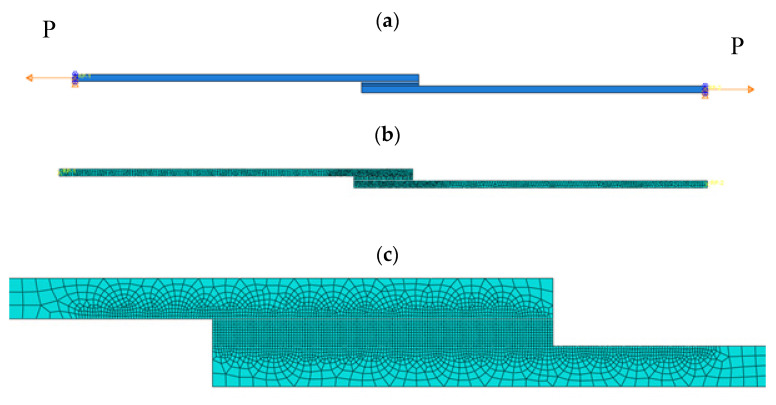
A specimen for numerical calculations: (**a**) manner of loading, (**b**) distribution of a finite element grid, (**c**) form of a grid of finite elements within the adhesive joint.

Nodes were deprived of the possibility of displacement and turning in axes perpendicular to the long specimen on the contact surface of the jaws of the strength testing machine (Figure 13a). Loading was applied through the displacement of flat bars, which was carried out symmetrically in both directions of the joint tension. Both the fixing and the displacement were applied to the specimen through reference points connected to the joint edge, through tying of the pivot type. Input data indicated the need to record the values of displacements, stress and strain for every 1% until 100% of the assigned displacement was achieved. This allowed the mapping of the whole of the lap joint tension process.

A division grid of the lap specimen is presented in Figure 13b. Finite elements of the CPE4R type were used: a 4-node bilinear plane strain quadrilateral, reduced integration and hourglass control. The shape of the finite elements in the adherends (flat bars of the specimen) is Quad-dominated and, in the adhesive, it is Quad. The elements in the adhesive were squares of size 0.1 mm along each side (Figure 13c). The size of the elements was selected through determination of the 2% error discretisation in two successive steps. In the zones of the adherend materials adjacent to the joint, the local density of the grid was consistent with the size between the nodes and was 0.1 mm.

## 3. Test results

### 3.1. Material Tests of Methacrylic Adhesive

**Figure 14:**
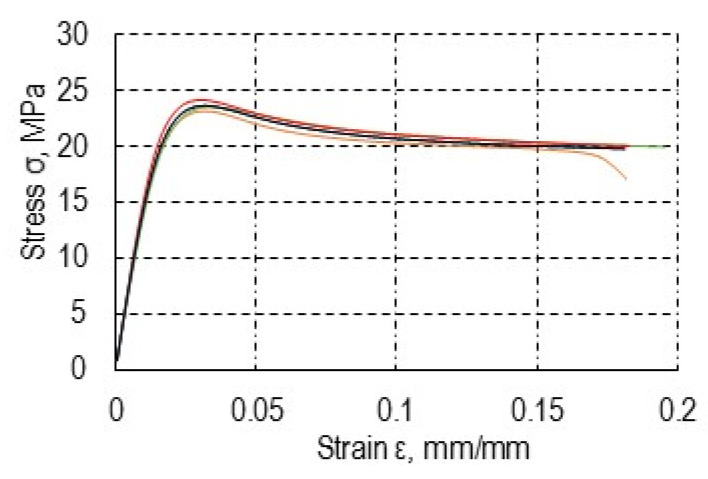
Stress diagram as a function of strain of the Plexus MA300 methacrylic adhesive.

The graphs in Figure 14 present examples of stress as a function of longitudinal strain for the tested material. Each of the specimens was destroyed in the tensile test. Specimens of methacrylic adhesives sustained significant transverse deformations, which made continuation of the test unnecessary. The yield point can be determined in the form of stress values at which the deformation occurs without a further load increase. Table 3 presents the average results of the testing of the mechanical properties of the Plexus MA300 structural methacrylic adhesive. The data specified by the manufacturers are given in brackets.

**Figure 15:**
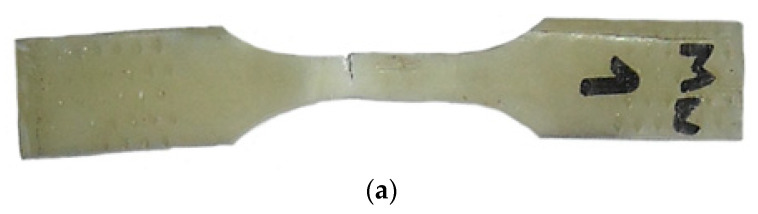

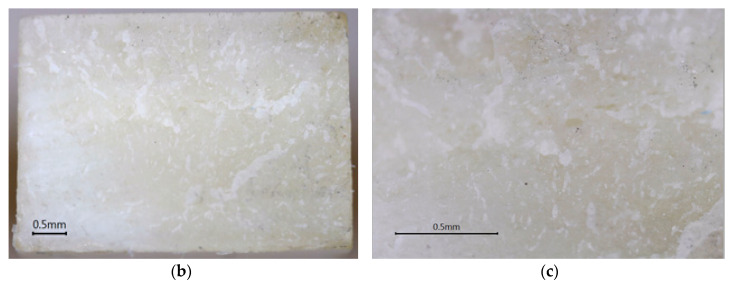
An example of specimen damage during tension: (**a**) view of the whole specimen, (**b**) view of the whole fracture, (**c**) zoom of the fracture fragment.

Figure 15a shows an example of a damaged specimen exposed to tensile loading. A rough analysis shows that plasticisation is characterised by a change of the specimen material colour, from transparent yellow to non-transparent white. The effect of necking is seen throughout the specimen length.

A crack occurred in the plane, perpendicular to the tensile direction. Plastic strain was also found in the cross-section of the specimen fracture. Figure 15b,c depict a change in the colour of the specimen tested, partly caused by the material strain effect due to tension. The white colour corresponds to those areas which underwent strong plastic strain. It indicates a differentiation of the localised properties of the tested material.

Based on the experimental tests, it is possible to state that the tensile strength and the value of the experimentally determined prolongation fit into the range of values specified by the manufacturer. In the case of Young’s modulus, the experimentally determined value is significantly higher than the value specified by the manufacturer (Table 1). In further parts of the study, experimentally determined properties of the adhesive will be used.

### 3.2. Test Results of Adherent Materials

**Figure 16:**
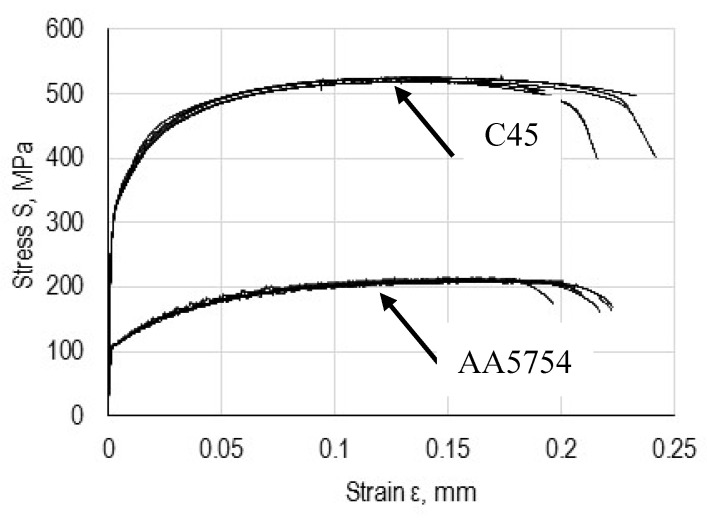
Stress diagrams as a function of strain of steel C45 and aluminium alloy 5754.

The graphs in Figure 16 present examples of stress as a function of longitudinal strain for adherend materials. Based on the graph, mechanical properties were determined and averaged and are presented in Table 4. The course and data of the tensile diagram were used for an analytic and numerical analysis of single-lap joints.

### 3.3. Test Results of Single-Lap Joints

**Figure 17:**
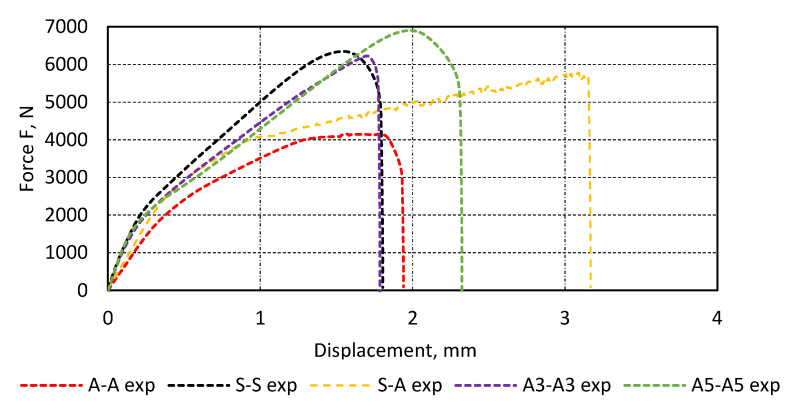
Exemplary test results for single-lap joint specimens.

The test results are presented in the form of a graph of force as a function of displacement for exemplary specimens of each type (Figure 17). The highest strength was characteristic of the 1.5 mm thick flat steel bar specimens and 1.5 and 5.0 mm thick aluminium ones. The lowest strength was found for the joints of aluminium and flat steel bars of 1.5 mm thickness. The results of all the tested specimens are presented in Table 5, including the mean value and standard deviation for a given type of specimen.

**Figure 18:**
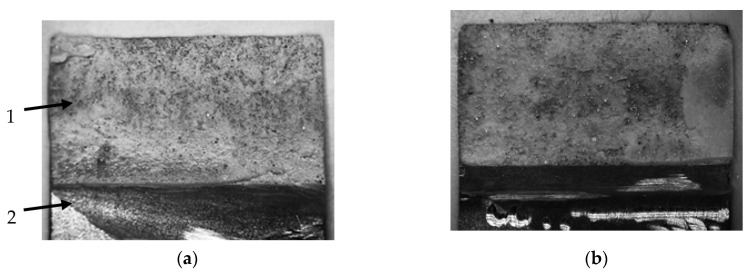

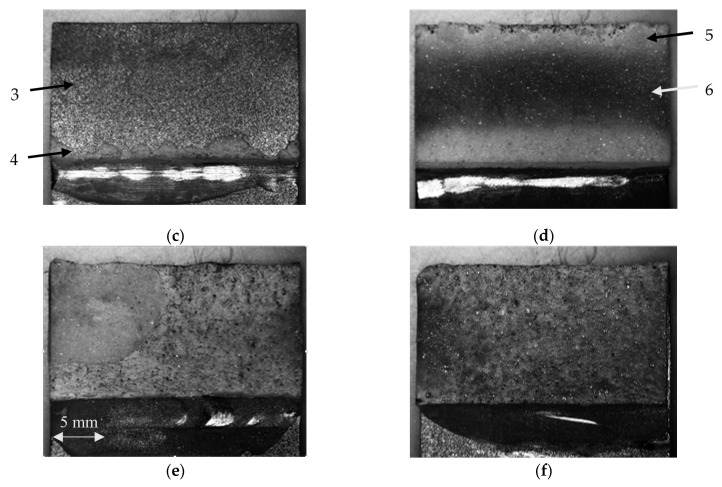
Examples of lap joint failure: (**a**) type A-A exp, (**b**) type S-S exp, (**c**) type S-A exp (aluminium side), (**d**) type S-A exp (steel side), (**e**) type 3-A3 exp, (**f**) type A5-A5 exp.

Figure 18 shows examples of the damage to specimens caused by an axial force. In Figure 18a, “1—joint” and “2—adherend” are marked. An analysis of the fractures indicates that the cohesion damage type prevailed in cross-sections of specimens of the same adherends: A-A, S-S, A3-A3, A5-A5. Remains of the adhesive were found on both adherends. The adhesive became white and non-transparent and this was observed in the entire sample. In the cross-section of the specimen where different materials were used (S-A), the adhesion failure mechanism was prevailing and is marked as “3” in Figure 18c. The failure cohesion mechanism that is only seen on one end of the lap is marked as “4” in Figure 18c. Almost the whole layer of the adhesive was left on the second element (Figure 18d). However, the adhesive colour change is visible on both ends of the lap (marked as “5”). This means that the adhesive only underwent plastic strain in those places. Stresses must have definitely been lower in the middle of the joint length because no colour change of the adhesive was found (marked as “6”).

### 3.4. Results of Stress and Strain Analysis

#### 3.4.1. Results of Analytic Methods

**Figure 19:**
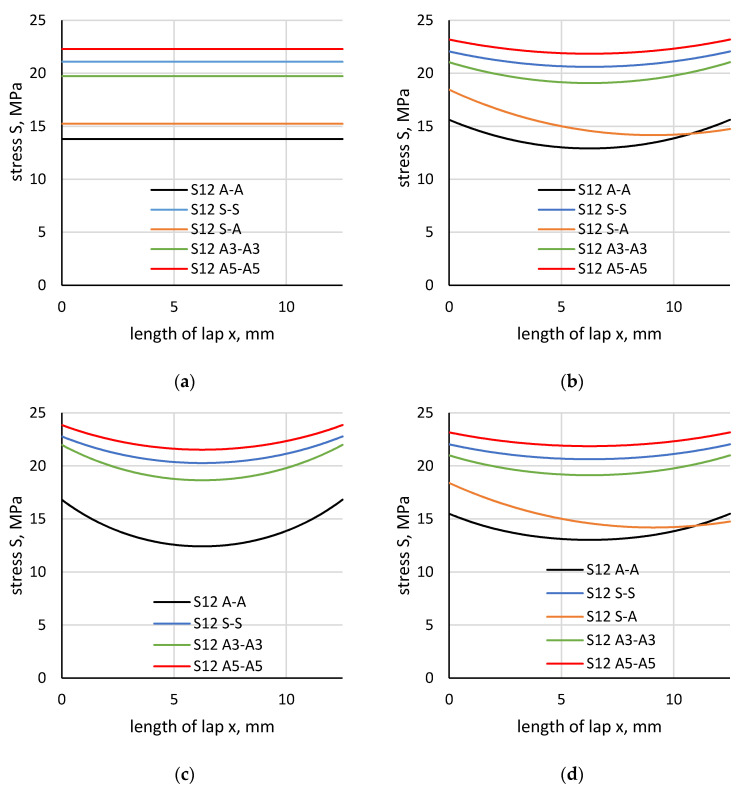
Distribution of stress determined by analytic methods: (**a**) engineering method, (**b**) Volkersen method, (**c**) Goland–Reissner method, (**d**) Adams–Pepiatt method.

The results of the stress distribution determination by analytic methods for values of forces that damage a given type of joint are presented in the graphs in Figure 19. The engineering method does not take into account the stiffness of the adherend materials and does not explain the differences between the resulting strengths of the joints. According to the Volkersen, Goland–Reissner and Adams–Pepiatt methods, a decrease in stiffness is accompanied by an increase in the non-uniformity of the stress distribution in the adhesive layer. The coefficient of the joint stress distribution non-uniformity is obtained by dividing the ratio of maximal values. The values of maximal stresses and coefficients calculated for each method are presented in Table 6. The coefficient reaches its highest value (i.e., about 1.2) for S-A and A-A joints and yet, the difference in the determined maximal shear stresses is still very high. This may indicate that stresses that occur in real joints were not well-reflected by the analytical methods or that the shear stress criterion accepted for the determination of the adhesive lap joint was incorrect.

#### 3.4.2. Results of Numerical Methods

**Figure 20:**
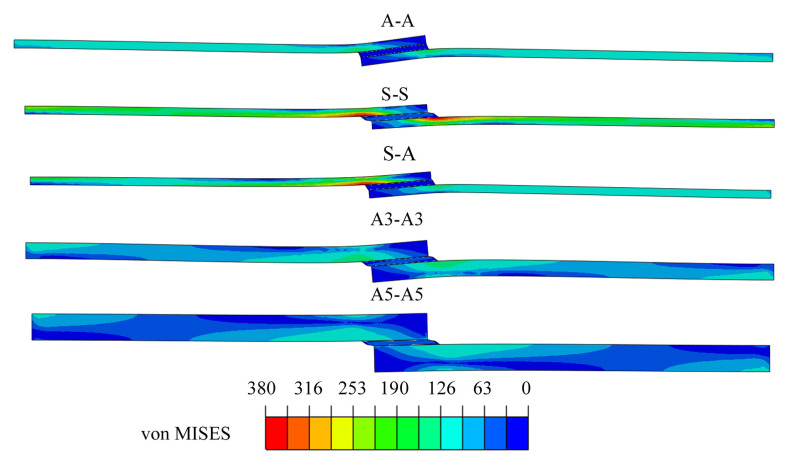
The form of strain of single-lap joints under the impact of loading force determined by the numerical method.

Strained forms of the specimens loaded by a failure force are presented in Figure 20. The strained forms of specimens are similar to the strain observed during experimental testing. The largest flexure was found for the least bend-resistant specimen (A-A), whereas the smallest flexure was visible in the stiffest specimens (S-S and A5-A5).

**Figure 21:**
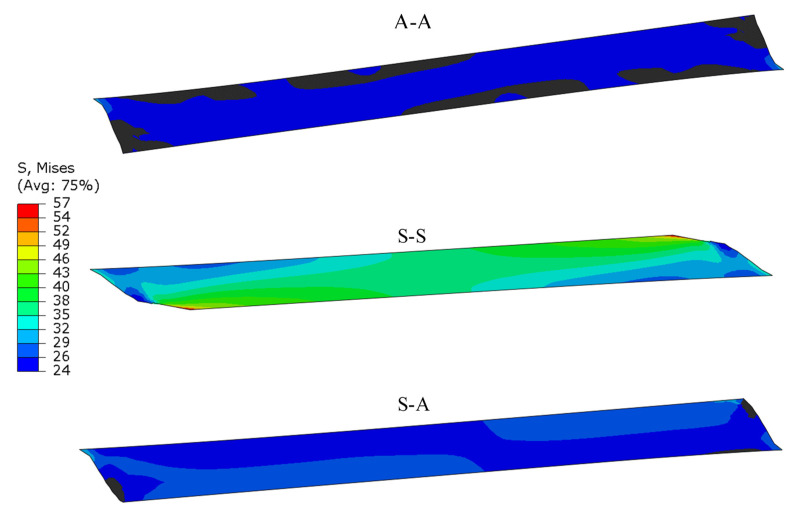
Map of the adhesive layer reduced stress, according to von Mises hypothesis.

Stress reduction in the layer of adhesive (according to the von Mises hypothesis) is shown in Figure 21. Significantly higher stress values can be observed for stiff elements (S-S) than those for less stiff elements (A-A). Along with an increase in the specimen stiffness, its ability to carry higher reduced loads increases (S-A). Similar results were found for other tensile hypotheses, e.g., Treska’s and other principal stresses. This can be caused by the fact that the influence of component stresses on the applied adhesive strength is different than that in the hypothesis used in the study.

**Figure 22:**
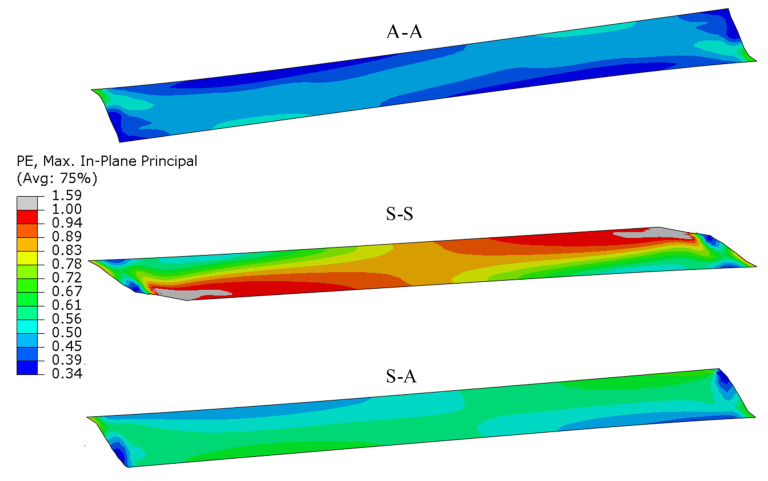
Map of the plastic strain component in the layer of the adhesive.

Components of plastic strain in the layer of the adhesive are shown in Figure 22. As in the case of reduced stresses, plastic strains in the layer of the adhesive differ depending on the stiffness of the bonded elements. Values higher than those for less stiff joints (A-A) can be observed for specimens (S-S). Hence, values of plastic strain are not a good strength criterion for specimens with different stiffness.

**Figure 23:**
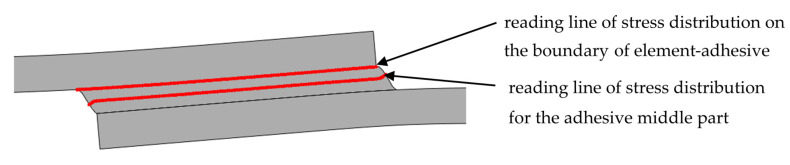
Localisation of the reading line of stress distribution on the boundary of element–adhesive and half length of the adhesive thickness.

**Figure 24:**
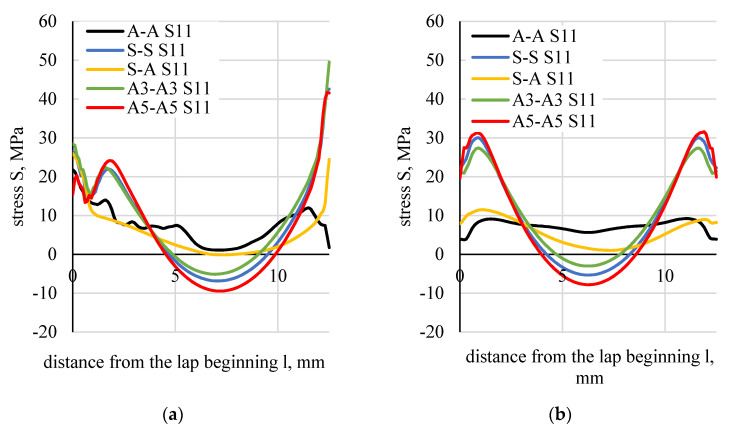
Distributions of normal stresses (S11) parallel to the direction of tension: (**a**) in the element–adhesive layer, (**b**) half length of the adhesive thickness.

**Figure 25:**
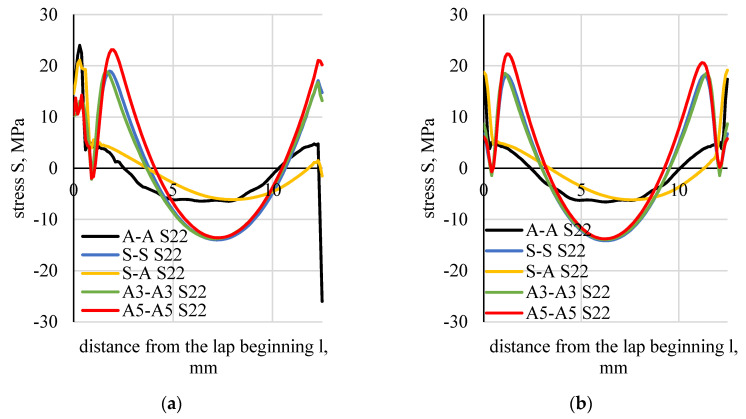
Distributions of normal stresses (S22) perpendicular to the direction of tension: (**a**) in the boundary element–adhesive layer, (**b**) half length of the adhesive thickness.

**Figure 26:**
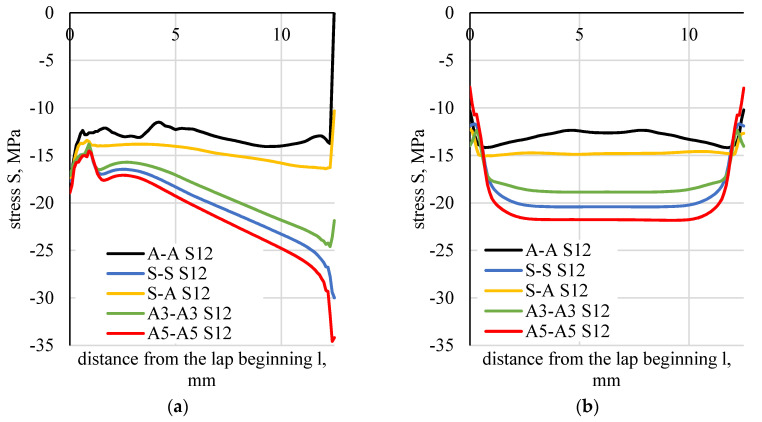
Distributions of shear stresses (S12): (**a**) in the boundary element–adhesive layer, (**b**) half length of the adhesive thickness.

**Figure 27:**
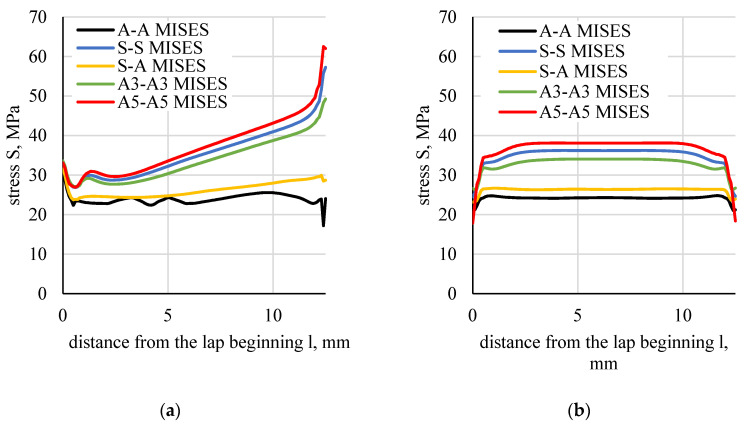
Distributions of stresses, reduced according to the Huber–Mises hypothesis: (**a**) in the boundary element–adhesive layer, (**b**) halfway of the adhesive thickness.

Stress distributions along the boundary of the element (flat bar), in the adhesive and in the middle of the adhesive thickness were determined based on the numerical calculations of lap joint strength (Figure 23). Figure 24 shows a distribution of normal stress determined in the direction parallel to the direction of tension (stresses were marked as S11). Figure 25 shows a distribution of normal stresses, determined along the direction perpendicular to the direction of tension (stresses are marked as S22). Figure 26 shows a distribution of shear stresses (stresses are marked as S12) and Figure 27 shows a distribution of stresses, reduced according to the Huber–Mises hypothesis.

**Figure 28:**
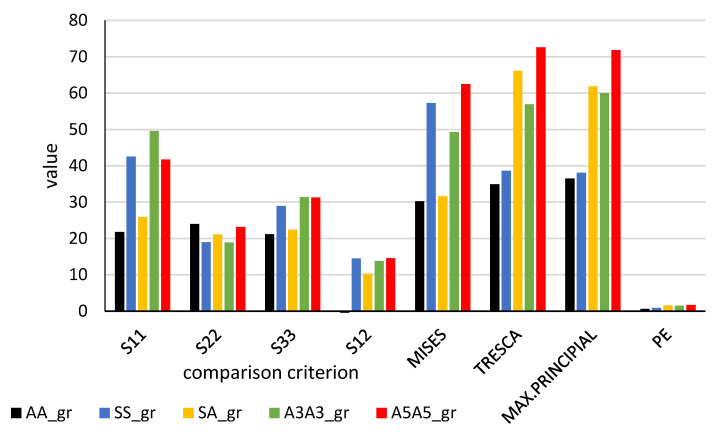
Comparison of maximal values of the potential failure criteria for the analysed types of joints (results for adhesive–element boundary layer).

**Figure 29:**
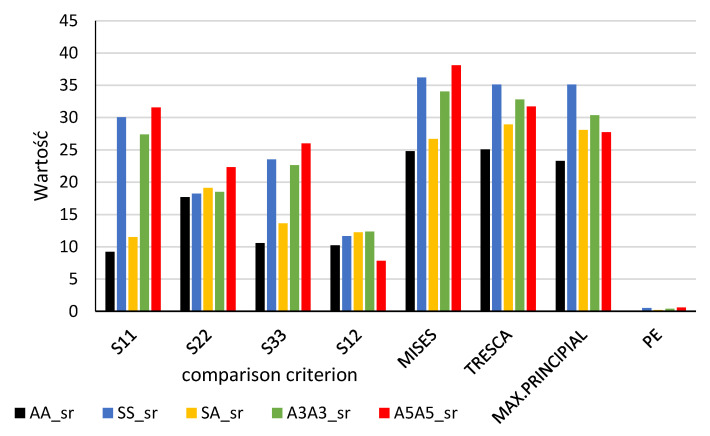
Comparison of the maximal values of potential failure criteria for the considered types of joints (results for the adhesive middle part).

Maximal values of the calculated distributions for the element–adhesive boundary layer are presented in the form of column diagrams (Figure 28) and the same for the layer halfway of the adhesive thickness (Figure 29).

#### 3.4.3. Comparison of Results from Analytical and Numerical Methods

The numerical method indicated that stress values were not identical, not only for the joint length, but also for its thickness. The boundary layer is characterised by significantly higher stresses. In connection with this, the layer was subjected to a more thorough strength analysis. In order to compare analytic and numerical results and find a criterion for the calculation of the joint failure force, the maximal values calculated for the boundary adhesive–element layer are presented in Table 7 and Table 8 and, for the adhesive middle layer, in Table 9.

#### 3.4.4. Comparison of Analytic and Numerical Method Test Results

Values of the boundary layer shear stresses (S12) were 1.5 times higher than those found in the middle layer. The values of S12 stresses, calculated analytically, assumed lower values than those calculated numerically. The difference increased along with the stiffness of the flat bar adherend materials.

The maximal and minimal values of the compared parameters were determined for five types of tested specimens (AA, SS, SA, A3A3, A5A5). The difference between the maximal and minimal value was divided by the maximal value. The ratio was expressed in %. The lowest values of this ratio were observed for normal stresses perpendicular to the direction of loading (S22), both in the adhesive boundary and the middle layers. None of the applied tensile hypotheses or the strain plastic component provided more consistent results for any of the tested specimens.

With regards to analytic methods, it was the Goland–Reissner method that provided the most consistent shear stress results. The highest difference was 30%. However, this method cannot be used for the calculation of elements with different stiffness. Maximal stresses (S12) determined by the Volkersen and Adams’s methods differ maximally by 33%, whereas those determined by the engineering method differ by 38%.

## 4. Conclusions

The paper compares several selected methods of predicting the strength of adhesive joints made of a methacrylic adhesive. In addition, the method of obtaining specimens and the results of material tests of the methacrylic adhesive and single-lap joints were presented.

The high standard deviation in the tests of the strength of single-lap joints is caused by many factors that affect the final result. The number of errors that may occur is demonstrated by the multi-stage, complicated process of preparing specimens described in the article.

Comparison of the consistency of the numerical method with the results of selected criteria indicated that the highest scatter was observed for normal stresses perpendicular to the direction of tension (S22). The biggest difference between the types of joints was 21% in this criterion. The results were much better than those obtained using the analytic methods of Goland–Reissner.

In addition to the inaccuracy of a given method of estimating the strength of joints, the results could also be influenced by the calculation of the average destructive force value for various types of joints. These values could be affected by a test error.

In a literature review, many researchers indicated different criteria for the failure of adhesive joints. The tested methacrylic adhesive is stretchable and deforms on average 19.4% before tensile rupture. Epoxy adhesives are less flexible but have more strength. Polyurethane adhesives have more flexibility but less strength. This may mean that different failure criteria should be used for the calculation of joints made of different types of adhesives.

The stiffness of the tested and simulated adherend materials of single-lap joints was variable. This significantly affected the type and the level of stress that occurred inside the layer of the adhesive which is confirmed by both analytical and numerical methods.

Use of a digital image correlation method for the measurement of the adhesive layer stress could be the next step. If there are differences between the simulation and experimental results, the adhesive material data may need to be corrected.

The analyses indicate that the most accurate method for assessment of the joint failure force to be used in design of single-lap adhesive joints is the method based on the value of normal stresses perpendicular to the direction of tension. It is necessary to take into consideration a significant error of the method which exceeds 20%.

## Figures and Tables

**Table 1:** Adhesive properties specified by the manufacturer [29].

Adhesive Properties	Tensile Strength	Young’s Modulus Ek	Strain to Failure	Lap Shear (ASTM D1002)
	MPa	MPa	%	MPa
Data from the adhesive manufacturer	20 to 24	931 to 1137	15 to 25	15 to 25

**Table 2:** Types of tested joints.

Specimen Marking	Materials and Their Thickness		
	First Element	Second Element	Adhesive
S-S	Steel C45 δ1 = 1.5 mm	Steel C45 δ2 = 1.5 mm	Plexus MA300 δk = 1 mm
S-A	Steel C45 δ1 = 1.5 mm	Aluminium 5754 δ2 = 1.5 mm	
A-A	Aluminium 5754 δ1 = 1.5 mm	Aluminium 5754 δ2 = 1.5 mm	
A3-A3	Aluminium 5754 δ1 = 3.0 mm	Aluminium 5754 δ2 = 3.0 mm	
A5-A5	Aluminium 5754 δ1 = 5.0 mm	Aluminium 5754 δ2 = 5.0 mm	

**Table 3:** Mechanical properties determined for the tested adhesives.

Mechanical Properties	Tensile Strength	Yield Strength	Young’s Modulus Ek	Poisson Ratio νk	Strain to Failure
	MPa	MPa	MPa	-	%
Mean value	23.7	23.7	1610.5	0.4	19.4
Standard deviation	0.4	0.4	52.9	0.02	0.9

**Table 4:** Mechanical properties of adherend materials.

Material	Mechanical Properties				
	Young’s Modulus	Poisson Coefficient	Tensile Strength	Yield Point	Prolongation until Rupture
	MPa	-	MPa	MPa	%
Steel C45	214,000	0.30	524.5	324.6	22.7
Aluminium Alloy 5754	74,000	0.34	213.0	107.8	21.4

**Table 5:** Test results for single-lap joint specimens.

Specimen Number	Lap Joint Specimen Strength—Maximal Nominal Shear Stresses (MPa)				
	A-A exp	S-S exp	S-A exp	A3-A3 exp	A5-A5 exp
Median	13.8	21.09	15.24	19.73	22.29
Standard deviation	0.91	1.22	0.94	1.28	0.93

**Table 6:** Maximal values of shear stress and stress distribution non-uniformity coefficients calculated analytically for failure forces obtained from the experiment.

Specimen Marking	Distribution Maximal Stresses				Coefficient of Stress Non-Uniformity Distribution		
	Engineering Method	Volkersen	Goland–Reissner	Adams	Volkersen	Goland–Reissner	Adams
A-A	13.80	15.61	16.81	15.56	1.13	1.22	1.13
S-S	21.09	22.06	22.78	22.05	1.05	1.08	1.05
S-A	15.24	18.45	-	18.39	1.21	-	1.21
A3-A3	19.73	21.04	21.99	20.97	1.07	1.11	1.06
A5-A5	22.29	23.19	23.85	23.10	1.04	1.07	1.04

**Table 7:** Maximal shear stress values of adhesives on the basis of analytical methods.

Specimen Marking	Analytic Methods			
	Engineering Method	Volkersen	Goland Reiss.	Adams
AA_gr	13.80	15.61	16.81	15.56
SS_gr	21.09	22.06	22.78	22.05
SA_gr	15.24	18.45	-	18.39
A3A3_gr	19.73	21.04	21.99	20.97
A5A5_gr	22.29	23.19	23.85	23.10
MAX	22.29	23.19	23.85	23.10
MIN	13.80	15.61	16.81	15.56
DIFFRENCE %	0.38	0.33	0.30	0.33

**Table 8:** Maximal values of the boundary layer of adhesive–element on the basis of FEM.

FEM							
Specimen Marking	S12	S11	S22	MISES	TRESCA	MAX PRINCI.	PE
AA_gr	17.21	21.78	**23.98**	30.24	34.92	36.50	0.61
SS_gr	29.99	42.55	**18.96**	57.25	38.59	38.09	0.86
SA_gr	17.34	25.92	**21.11**	31.59	66.10	61.86	1.59
A3A3_gr	24.58	49.56	**18.90**	49.26	56.90	60.00	1.50
A5A5_gr	34.57	41.72	**23.13**	62.52	72.62	71.85	1.71
MAX	34.57	49.56	**23.98**	62.52	72.62	71.85	1.71
MIN	17.21	21.78	**18.90**	30.24	34.92	36.50	0.61
DIFFRENCE %	1.01	0.56	**0.21**	0.52	0.52	0.49	0.64

**Table 9:** Maximal distribution values in the adhesive middle layer on the basis of FEM.

FEM							
Specimen Marking	S12	S11	S22	MISES	TRESCA	MAX PRINCI.	PE
AA_sr	14.19	9.20	**17.69**	24.80	25.09	23.31	0.01
SS_sr	20.43	30.05	**18.22**	36.20	35.14	35.10	0.52
SA_sr	15.07	11.49	**19.14**	26.69	28.92	28.06	0.20
A3A3_sr	18.87	27.39	**18.52**	34.03	32.81	30.37	0.39
A5A5_sr	21.84	31.57	**22.34**	38.10	31.71	27.72	0.60
MAX	21.84	31.57	**22.34**	38.10	35.14	35.10	0.60
MIN	14.19	9.20	**17.69**	24.80	25.09	23.31	0.01
DIFFRENCE %	0.54	0.71	**0.21**	0.35	0.29	0.34	0.98

## Abbreviation

l	length of lap
l_1_,l_2_	length of elements
b	width of joint
δ_1_, δ_2_	width of element 1, 2
δ_k_	width of adhesive layer
E_1_,E_2_	Young’s modulus of the material 1, 2
G_1_,G_2_	Kirchoff modulus 1, 2
ν_1_,ν_2_	Poisson modulus 1, 2
E_k_	Young’s modulus of adhesive
G_k_	Kirchoff modulus of adhesive
ν_k_	Poisson modulus of adhesive
P	force loading joint
γ	shape strain
ε	linear strain
τ	shear stress
σ	normal stresses
